# *Punica granatum* L. Hydrogel for Wound Care Treatment: From Case Study to Phytomedicine Standardization

**DOI:** 10.3390/molecules21081059

**Published:** 2016-08-22

**Authors:** Aline Fleck, Patrik F. G. Cabral, Felipe F. M. Vieira, Deo A. Pinheiro, Carlos R. Pereira, Wilson C. Santos, Thelma B. Machado

**Affiliations:** 1 Programa de Pós-Graduação em Ciências Aplicadas a Produtos Naturais, Universidade Federal Fluminense, Rua Dr. Mario Viana, 523, Santa Rosa, Niterói, RJ, CEP: 24230-540, Brazil; alinefleck@hotmail.com (A.F.); wsantos@id.uff.br (W.C.S.); 2 Faculdade de Farmácia, Universidade Federal Fluminense, Rua Dr. Mario Viana, 523, Santa Rosa, Niterói, RJ, CEP: 24230-540, Brazil; patrik_gandara@hotmail.com (P.F.G.C.); felipefelizardo@id.uff.br (F.F.M.V.); deo2@ig.com.br (D.A.P.); 3 Programa de Pós-Graduação em Engenharia de Biossistemas, Universidade Federal Fluminense, Rua Passo da Pátria, 156, Bloco D, sala 236, São Domingos, Niterói, RJ, CEP: 24210-240, Brazil; crpereira@vm.uff.br

**Keywords:** *Punica granatum*, wound healing, hydrogel, punicalagin, quality control

## Abstract

The pharmacological activities of many *Punica granatum* L. components suggest a wide range of clinical applications for the prevention and treatment of diseases where chronic inflammation is believed to play an essential etiologic role. The current work reports a case study analyzing the effect produced by a magistral formulation of ethanolic extracts of *Punica granatum* peels on a non-healing chronic ulcer. The complete closure of the chronic ulcer that was initially not responsive to standard medical care was observed. A 2% (*w*/*w*) *P. granatum* peels ethanolic extract hydrogel-based formulation (PGHF) was standardized and subjected to physicochemical studies to establish the quality control parameters using, among others, assessment criteria such as optimum appearance, pH range, viscosity and hydrogel disintegration. The stability and quantitative chromatographic data was assessed in storage for six months under two temperature regimes. An efficient HPLC-DAD method was established distinguishing the biomarkers punicalin and punicalagin simultaneously in a single 8 min run. PGHF presented suitable sensorial and physicochemical performance, showing that punicalagin was not significantly affected by storage (*p* > 0.05). Formulations containing extracts with not less than 0.49% (*w*/*w*) total punicalagin might find good use in wound healing therapy.

## 1. Introduction

Herbal formulations have been recommended for the management of skin properties for a long time and their effects are well accepted by modern society, also resulting in good consumer acceptance [1,2]. Crude aqueous or ethanolic herbal extracts have been used to treat various skin ailments such as wounds, psoriasis and inflammatory conditions [3]. They are also important sources of biologically active natural products and have been extensively used in pharmaceutical industry research and development studies that have resulted in the identification of compounds with therapeutic potential [4].

*Punica granatum* L. (Punicaceae), commonly known as pomegranate, is a shrub or a small tree native to the Mediterranean region. The different parts of pomegranate (*Punica granatum* L.) have been known as a reservoir of bioactive compounds with potential biological activities [5]. This species is rich in phenolic compounds and is an important source of hydrolysable tannins, ellagitannins and ellagic acid [6]. In addition to the tannins, pomegranates also contain flavonoids, including flavones, flavonols and anthocyanidins. Epicatechin, epigallocatechin and their derivatives are also present in pomegranates. The brilliant red colour is attributed to anthocyanins, which are the major constituents of arils [7]. Pomegranate fruit juice, fruit and peel extracts have been found to possess a remarkable antioxidant activity [8]. Further, its antibacterial, anticonvulsant, antiinflammatory, antifungal, immunomodulatory, cardioprotective, antimutagenic, antispasmodic and antidiabetic activities have also been documented [6].

*Punica granatum* peel extract was evaluated for its wound-healing activity in rats using an excision wound model [9]. The authors have shown that the extract-treated animals were found to epithelialize faster when compared to respective controls and exhibited a 95% reduction in the wound area. These extracts were also investigated for healing effects on deep second-degree burns in rats [10]. Histological results indicated that inflammatory cells substantially disappeared and were replaced by new granulation tissue in extract treatment, while that of the control rats still showed severe inflammatory cell infiltration. In vivo potential of *P. granatum* peels was also described for a 5% (*w*/*w*) methanolic extract based-ointment formulated and evaluated for its wound healing in guinea pigs. The ointment significantly enhanced wound contraction, period of epithelialization and also the biochemical and histopathological characteristics [11].

**Figure 1:**
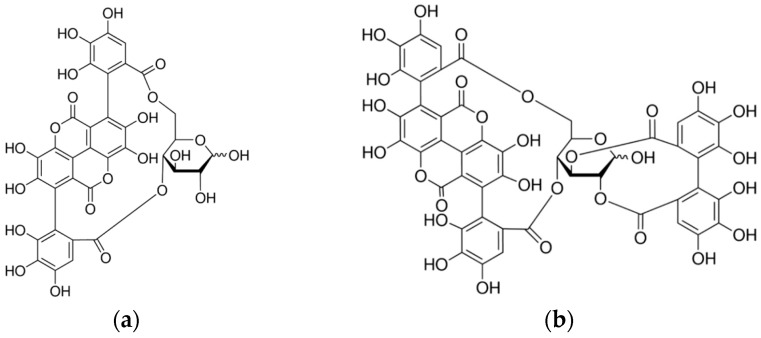
Chemical structures of the ellagitannins punicalin (**a**) and punicalagin (**b**).

In a previous work, the ellagitannins punicalin and punicalagin (Figure 1a,b) were isolated from the hydroalcoholic extracts of *P. granatum* peels and punicalagin was found to be responsible for the antimicrobial activity of the extract against sensitive and multi-drug resistant bacteria, especially species of staphylococci, such as *Staphylococcus aureus* [12]. Punicalagin has also been reported to have a wide range of biological effects, including antioxidant, hepatoprotective, antimicrobial, antiproliferative, apoptotic, chemopreventive and immune-suppressive activities [13].

Polyphenols are known for their ability to precipitate proteins, which is one of the supportive clues for their wound healing properties [14]. Due to the high rates of recurrence of chronic wounds, new effective therapeutic strategies need to be developed. Plants represent important sources of alternative formulations that will complement the skin therapy arsenal in either prevention or treatment. Despite the promise of *P. granatum* extracts as a therapeutically active ingredient for many applications, there is little data available concerning the standardization and stability of extracts that would potentially be suitable for the development of an appropriate topical preparation for use in clinical trials. In order to find a suitable biomarker to establish quality control parameters of *P. granatum* peel extracts and upon consideration of its physiochemical properties and stability, a hydrogel-based formulation was developed and evaluated.

### Case Presentation

**Figure 2:**
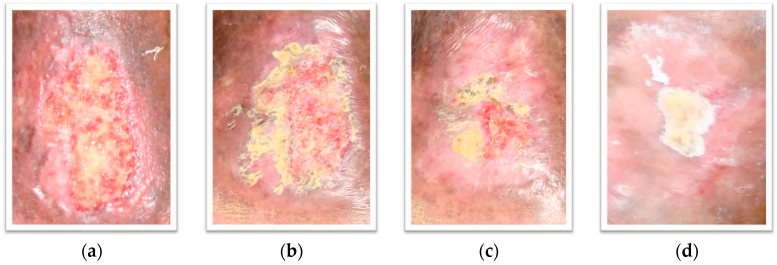
Photographs of the patient’s wound. PGMF: *Punica granatum* magistral formulation; (**a**): 1 day before PGMF treatment; (**b**): week 1 of PGMF treatment; (**c**): week 6 of PGMF treatment; (**d**): week 10 of PGMF treatment.

A 76-year-old woman presented with the chief complaint of a non-healing ulcer on the left leg, with pain and swelling. The ulcer had not responded to consistent conventional treatment for more than one year. The patient was a non-smoker and non-dinker. There was no history of trauma, varicose veins, or calf pain, but a history of vascular disease. Physical examination revealed a single shallow, irregular, large, almost rectangular shaped, about 6.8 × 4.3 cm ulcer on her lower left leg. The surface of the ulcer demonstrated shiny granulation tissue (Figure 2a). There were mild eczematous changes of the surrounding skin. There was a purulent foul smelling moderate amount of discharge from the wound, with surrounding edema. The patient reported pain at the affected area while walking and was referred to the angiology service for wound management. Despite rigorous medical measures in the form of frequent use of anabolic and topical antimicrobial agents (clostebol, neomycin, gentamicin and silver sulphadiazine), topical corticosteroids (betamethasone dipropionate), debridement, and dressing for the ulcer, the subject had a recurrent chronic wound on the left leg with an area measuring 23.52 cm^2^. The ulcer was in stage II, with not well defined wound margins. The angiologist prescribed a magistral formulation of a 2% (*w*/*w*) *Punica granatum* peel ethanolic extract (PGMF) based on a hydrophilic cream and zinc oxide. PGMF was applied to the ulcer once a day and the physician’s decision of application was taken with the written informed consent of the patient. Oral iron therapy, diuretics and other supplementary treatment were initiated. Tramadol was prescribed as pain reliever and no antibiotics were given during this course of treatment. After application, the ulcer was dressed with cotton gauge. Within six weeks, the ulcer had decreased to one quarter of its original size and had completely healed six weeks later (Figure 2b–d). A total of 90 applications were needed for complete healing. No adverse effects of PGMF were observed.

## 2. Results

### 2.1. Method Validation

**Figure 3:**
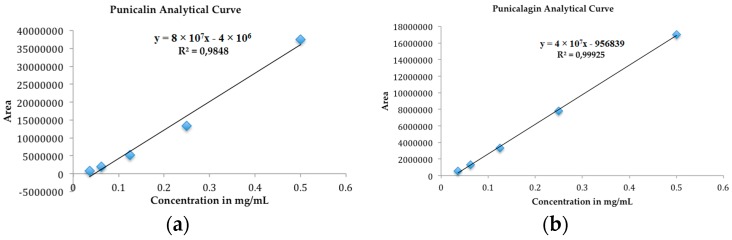
Analytical curves of the biomarkers (**a**) punicalin and (**b**) punicalagin.

**Figure 4:**
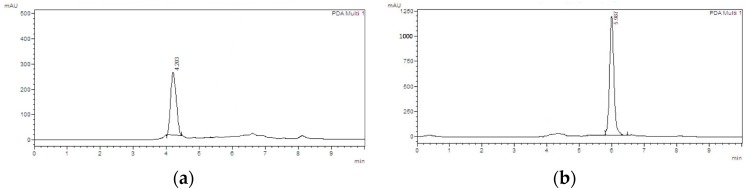
HPLC chromatographs of punicalin (**a**) and punicalagin (**b**) standards. Chromatograph was run on a Shimadzu LC-20AT HPLC equipped with a SPD-M20A PDA detection system. Separations were carried out on a Dr. Maisch GmbH C18 (Ammerbuch-Entringen, Germany) column (5 μm, 250 mm × 4.6 mm), 25 °C, with a mobile phase of 0.1% (*v*/*v*) aqueous solution of phosphoric acid and acetonitrile (80:20). The mobile phase flow rate was 1 mL/min with 10 min run time. The standards were detected using a wavelength of 254 nm. Sample injection volume was 10 μL. Retention times for punicalin and punicalagin were 4.203 and 5.987 min, respectively.

In order to validate the analytical method [15], five punicalin and punicalagin standard solutions were prepared at concentrations ranging from 0.5 to 0.0362 mg/mL (injection volume of 10 μL). Every sample was analyzed five times. The variance analysis (ANOVA) of the linear regression confirmed the linearity of the method, through rejection of the null hypothesis of linearity deviation for a significance level of 0.05; the coefficient of variation of the method was 5.2%. The equations of the regression lines, correlation coefficients and chromatograms are shown in Figure 3 and Figure 4a,b. The method precision (as repeatability) was 0.62%, as determined by a six-fold analysis of the same sample. System accuracy was expressed as percentage recovery by the assay of known added amounts of standards, showing a mean value of 102.9% (*n* = 9). The detection and quantitation limits (LOD and LOQ), based on the standard deviation of the response and slope were 0.023 and 0.077 mg/mL for punicalin and 0.011 and 0.037 mg/mL for punicalagin, respectively. The sensivity of the method was evaluated by determining LOD and LOQ, showing good linearity relationship in the concentration range between LOQ and 130 times LOQ for punicalin, and 270 times LOQ for punicalagin. All data set were statistically evaluated by ANOVA single factor, showing no significant difference at 95% confidence (*p* > 0.05). Specificity was evaluated by analyzing peak purity (angle and threshold) at retention time (RT) corresponding to punicalin and punicalagin. Data used were derived from the PDA workstation software resource (LCsolution workstation Multi-PDA 223-0560392, Shimadzu, Tokyo, Japan) for standards and PGHF samples analyzed in association with the software resource provided by the LC workstation (LCsolution workstation Postrun 223-05604-02, Shimadzu). The peak purity was determined using multivariate analysis by comparison of RT and peak area. A robustness test was performed to examine the effect of operational parameters on the analysis results. The flow-rate (0.7 ± 0.01 mL/min), injection volume (10 ± 0.5 μL), temperature (25.0 ± 0.5 °C) and mobile phase composition (80.0 ± 1/20.0 ± 0.2) were determined in order to confirm the robustness of the method.

### 2.2. Stability Tests and Sensorial Evaluation

#### 2.2.1. Spreadability, Centrifuge Accelerated Deterioration and Microbiological Analysis

**Figure 5:**
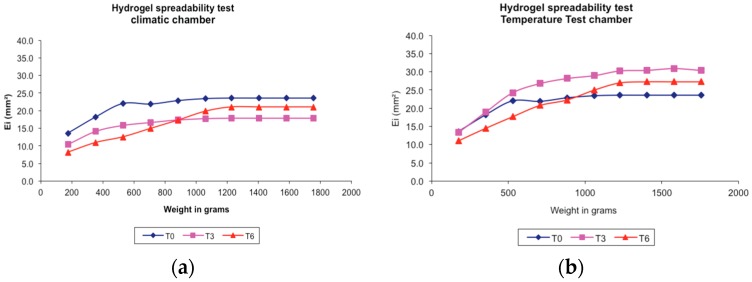
Spreadability test of hydrogel-based formulation of ethanolic extract of *P. granatum* peels (2% *w*/*w*) in different storage conditions. (**a**) Climatic chamber; (**b**) Temperature test chamber; T0: after production; T3: 3 months; T6: 6 months. Results are represented by means (*n* = 3).

*Punica granatum* peels ethanolic extract hydrogel-based formulation (PGHF) spreadability behaviors under different storage conditions are shown in Figure 5a,b. The centrifuge accelerated deterioration test is widely used as means to assess the long-term stability of hydrogels and creams. In terms of a typical ionic hydrogel-based formulation, the constituents have low tendency to separate avoiding break up and phase separation. The hydrogel formulation evaluated in this study has met the specifications of the test.

No significant results for microbiological analysis were found during the stability study. The microbiological evaluation showed the efficiency of manipulation techniques and microbiological preservatives to keep the formulation characteristics and prevent growth of microorganisms.

#### 2.2.2. pH Measurement

The human skin has a slightly acidic pH between 4.6 and 6.0 [16]. This pH range contributes to the protection of the skin surface against bacteria and fungi. Table 1 shows pH values for PGHF at T0, T3 and T6 under different packaging and storage conditions. Regardless of the different parameters evaluated, pH differences were not significant (*p* > 0.05), corroborating the null hypothesis (H_0_) and showing that the formulation remained within the ideal pH range for human skin.

#### 2.2.3. Viscosity

**Figure 6:**
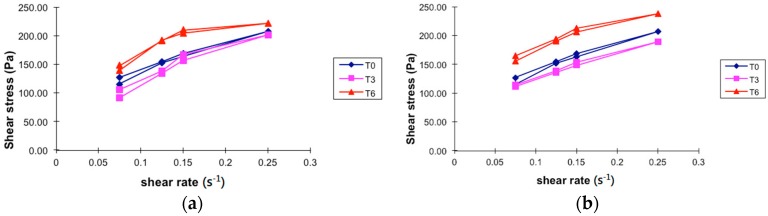
Share stress—share rate curves of hydrogel-based formulation of ethanolic extract of *P. granatum* (2%, *w*/*w*); T0: after production, T3: 3 months and T6: 6 months of storage under different storage conditions. (**a**) Temperature test chamber; (**b**) Climatic chamber. Results are represented by means (*n* = 3).

Rheograms for PGHF are shown in Figure 6a,b. The rheological properties of PGHF clearly showed that the slope (viscosity of the hydrogel) decreased as the shear rate increased, which indicated that the hydrogel presented pseudoplastic flow. In fact, with the increasing shear rate, the structure of the hydrogel began to disrupt and the particles started to align until it started flowing.

**Figure 7:**
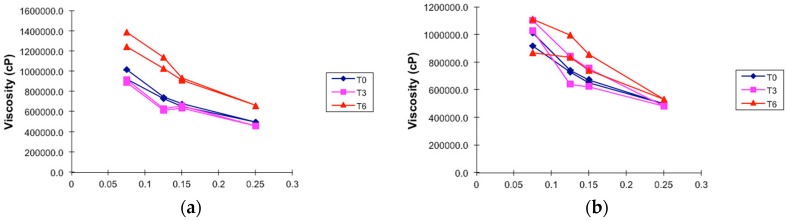
Viscosity—share rate curves of hydrogel-based formulation of ethanolic extract of *P. granatum* (2%, *w*/*w*); T0: after production, T3: 3 months and T6: 6 months of storage at different storage conditions. (**a**) Climatic chamber; (**b**) Temperature test chamber. Results are represented by means (*n* = 3).

On the other hand, PGHF showed a high viscosity (Figure 7a,b), which is suitable for topical formulation adherence to the skin. Besides, no obvious change in viscosity was observed after 6 months of storage at two different temperatures (temperature test chamber and climatic chamber), packaging (aluminum or polyethylene tube) and time (T3 and T6) compared with that of hydrogel at T0. All these results indicated that the formulation was stable and ensured the maintenance of non-Newtonian flow and also thixotropic and pseudoplastic behavior.

#### 2.2.4. Gel Disintegration Study

**Figure 8:**
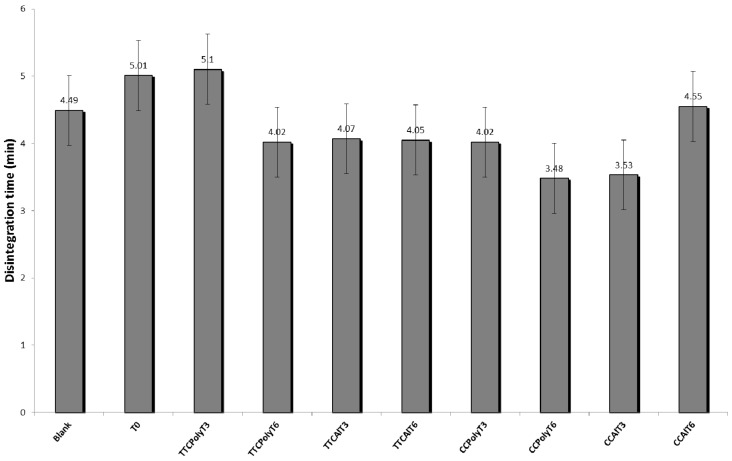
Disintegration time of hydrogel-base formulations. Blank; after production (T0); temperature test chamber-polyethylene-time: 3 months (TTCPolyT3); temperature test chamber-polyethylene-time: 6 months (TTCPolyT6); temperature test chamber-aluminum-time: 3 months (TTCAlT3); temperature test chamber-aluminum-time: 6 months (TTCAlT6); climatic chamber-polyethylene-time: 3 months (CCPolyT3); climatic chamber-polyethylene-time 3months (CCPolyT6); climatic chamber-aluminum-time: 3 months (CCAlT3); climatic chamber-aluminum-time: 6 months (CCAlT6). Results are represented by means ± S.E.M. (*n* = 3).

Disintegration time of a hydrogel-based formulation (blank) and PGHF at different storage and packaging conditions are shown in Figure 8. Disintegration time data from the different samples analyzed are represented by the mean ± the standard deviation of triplicate samples. The results showed no significant difference (*p* > 0.05) in time for complete disintegration of hydrogel samples, even when stored in different packaging, time of storage and under more drastic storage conditions (accelerated stability tests). Time for complete disintegration of all samples was very low, between 3.5 and 5.1 min. These results showed that the concentration of gelling agent used in the formulation was adequate and that the topical phytomedicine formulated in the study can be easily removed.

#### 2.2.5. HPLC-DAD Analysis

**Figure 9:**
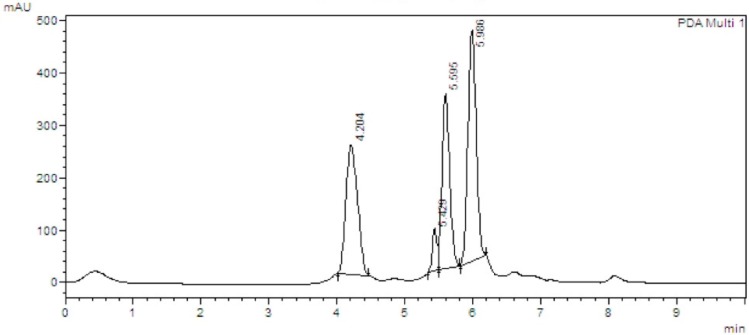
HPLC chromatographs of *Punica granatum* peel ethanolic extract hydrogel-based formulation (PGHF), showing retention times for punicalin (4.203 min) and punicalagin (5.987 min). Chromatography was run on a Shimadzu LC-20AT HPLC with SPD-M20A PDA detection. Separations were carried out on a Dr. Maisch GmbH C18 column (5 μm, 250 mm × 4.6 mm), 25 °C, with a mobile phase of 0.1% (*v*/*v*) aqueous solution of phosphoric acid and acetonitrile (80:20). The mobile phase flow rate was 1 mL/min with 10 min run time. The standards were detected using a wavelength of 254 nm. Sample injection volume was 10 μL.

**Figure 10:**
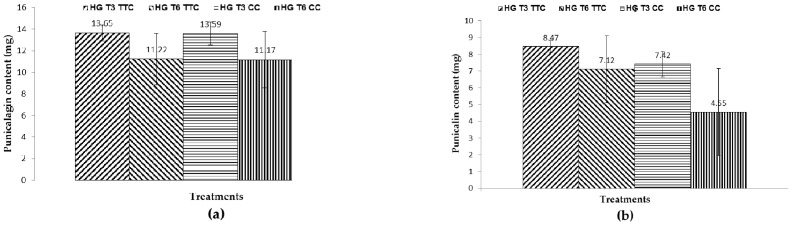
Punicalagin (**a**) and punicalin (**b**) contents in PGHF; HGT3TTC-hydrogel, time: 3 months, temperature test chamber; HGT6TTC-hydrogel, time: 6 months, temperature test chamber; HGT3CC -hydrogel, time: 3 months, climate chamber; HGT6CC-hydrogel, time: 6 months, climate chamber. Results are represented by means ± S.E.M. (*n* = 3). No statistical significant difference was detected. Statistical analysis was performed by two-way ANOVA followed by Bonferroni’s test of multiple comparisons (*p* < 0.05).

Quantification results for the biomarkers punicalin and punicalagin in PGHF are shown in Figure 9 and Figure 10. Punicalagin content showed no statistical difference (*p* > 0.05) when comparing packaging (aluminum or polyethylene tubes) and storage conditions (temperature test or climate chambers), corroborating the null hypothesis (H_0_). Table 2 shows the statistical significance levels in different correlations analyzed in this study.

The results showed that during three months of storage there was no significant difference of punicalagin content in the hydrogel formulation when the product was stored in a temperature test chamber or in a climatic chamber. There was a significant reduction of 12.4% in punicalin content when the product was stored in a climatic chamber (T3) compared with the product stored in a temperature test chamber. This reduction in punicalin content was also observed after six months (T6) in the product stored in a climatic chamber, showing a reduction of 38.7% compared to T3. Punicalagin content in the ionic hydrogel formulation showed no statistical difference between time and storage conditions (*p* > 0.05), showing that punicalagin has greater stability compared to punicalin.

## 3. Discussion

Polymeric hydrogels with ionic pendant groups can accept and/or donate protons in response to an environmental pH change [17]. In a pH-responsive hydrogel at a specific pH, the degree of ionization known as p*K*_a_ or p*K*_b_ is dramatically changed. This rapid change in the net charge of ionized pendant groups causes an abrupt volume transition by generating electrostatic repulsive forces between ionized groups, which creates a large osmotic swelling force. Two major factors control the degree of swelling of ionic hydrogels. The first factor is the property of the polymers such as ionic charge, concentration and p*K*_a_ or p*K*_b_ of the ionizable groups, degree of ionization, crosslink density as well as hydrophilicity or hydrophobicity. The second factor is the property of the swelling medium like pH, ionic strength and the counter ion and its valence [18]. Personal care formulations are typically formulated at a mildly acidic to a mildly alkaline pH, so it is important to understand how pH affects the rheological properties of hydrogels made with carbomers. The influence of the physicochemical parameters of aromatic molecules substituted at the stage of hydrogel was previously reported showing that the mechanism of interaction between hydrogel and substituted aromatic moiety is a complex sum of different molecular physicochemical properties that may influence polymer properties such as viscosity of the solution, dispersion and drug release [19]. Pharmacopoeia monographs and scientific references describe that non-ionic materials such as methylcellulose have incompatibilities with phenolic compounds [20,21]. The hydrogel-based formulations manipulated in this study showed ionic characteristics compatible with the phenolic compounds present in high concentrations in the ethanol extract of *P. granatum* peels. The presence of high concentrations of phenolic compounds and pH change associated with the final product, have led to different mechanisms of physicochemical interactions. This complexity of physical and chemical interactions between the carbomer used in the formulation and the complex mixture of phenolic molecules present in the extract resulted in an end product with particular characteristics.

In daily care related to the treatment of wounds there is a great concern about the techniques used for the removal of bandages and medicines applied to the lesion and, consequently, its sanitation. This process can be very painful for the patient, which means that special attention is necessarily given in the development of formulations exhibiting characteristics that make this process easier, minimizing patient discomfort and allowing the ready examination of the lesions [22]. According to USP regulations on semisolid formulations, the product quality attributes should include, but are not limited to the following: description, assay, uniformity, physicochemical properties and apparent viscosity. In addition, the appearance, tactile feeling, spreadability and odor of formulations are crucial properties, which closely relate to patient compliance. Furthermore, acceptability is determined with subjective assessment of appearance, tactile feel, texture and odor. The hydrogel-based formulation evaluated was found to give the optimal spreadability (scored “0”), acceptable color appearance, tactile feel, uniform texture and low-odor. No significant results for microbiological analysis were found during the stability study. The microbiological analysis evaluation showed the efficiency of manipulation techniques and microbiological preservatives to keep the formulation characteristics and prevent growth of microorganisms.

The ellagitannins punicalin and punicalagin were identified as the major chemical constituents of purified active fractions of peels extracts of *Punica granatum* [12]. The evaluation of the antimicrobial activity of isolated punicalagin against multiresistant hospital staphylococcal strains showed that this ellagitannin is the biomarker responsible for the inhibitory activity, showing the same Minimum Inhibitory Concentration (MIC) of active fractions (62.5 μg/mL). In the present investigation, a 2% (*w*/*w*) ethanolic extract ionic hydrogel formulation showed an average punicalagin content of 17.9 mg. Under more drastic storage conditions (climate chamber, 40 °C/75% RH), the average content of punicalagin reached 11.2 mg (37.4% reduction). However, the proposal of a formulation of 2% (*w*/*w*) was due to the fact that in a previous study a 1% (*w*/*w*) product had already been proposed, since the fractions enriched with ellagitannins isolated from hydroalcoholic extracts of *P. granatum* peels had the same MIC of micronized silver sulfadiazine (62.5 μg/mL) over the same multi-resistant bacteria [23]. Moreover, the results obtained in the case study were related to a 2% (*w*/*w*) formulation of a hydroalcoholic extract of *P. granatum* peels.

Silver sulfadiazine is a drug of choice for treating difficult to treat burns and wounds, both for its healing action and for its antimicrobial activity. Silver sulfadiazine products are usually prescribed as 1% (*w*/*w*) creams or ointments to 1%. The formulation evaluated in the present study has a double concentration of the product previously proposed, ensuring a longer shelf life for the product standardized with punicalagin as its biomarker. Further studies to determine the shelf life of the hydrogel-based formulation, long-term studies (24 months), and toxicological studies are necessary to be performed, so that the product will have guaranteed effectiveness and safety.

## 4. Materials and Methods

### 4.1. Chemicals

Punicalin and punicalagin standards were obtained from Biopurify Pharmaceuticals Ltd. (Chengdu, China), purity over 98%. HPLC-grade solvents and all other chemicals used in this study were obtained either from Merck Chemical Company (Darmstadt, Germany) or from Vetec (Rio de Janeiro, Brazil).

### 4.2. Plant Material and Preparation of the Extract

Fruits of *Punica granatum* L. were collected in the city of Nova Iguaçu, RJ, in June 2014. Peels were separated and dried in an oven with forced air circulation at 37 °C (Model 411D, Nova Ética, São Paulo, Brazil) and pulverized by a mechanical grinder. A voucher specimen (RFA No. 30906) was deposited in the Department of Botany herbarium at Universidade Federal do Rio de Janeiro. The powder (250 g) was macerated with 2 L of aqueous ethanol (80%) under shade. This procedure was repeated until exhaustion of plant materials. The extracts were filtered and concentrated using vacuum rotatory evaporation (85-01 LABTEC LB, São Paulo, Brazil) at 40 °C. The dried extracts (21.7% ± 0.76%, ethanol free) obtained were used for formulation of a 2% (*w*/*w*) hydrogel-based formulation using carbomer 980 (1%), imidazolidinyl urea (0.5%), methylparaben (0.15%), aminomethylpropane (1%) and distilled water up to 100 g.

### 4.3. Stability Tests and Sensorial Evaluation

The formulation was packaged in two different containers (aluminum and polyethylene tubes) and stored both in a climatic chamber at 40 ± 2 °C/75 ± 5% RH (Zone IV) and in a temperature test chamber at 30 ± 2 °C/ambient humidity (Nova Ética). Samples were evaluated in triplicate at 0 (T0), 3 (T3) and 6 (T6) months and the procedures were performed in accordance with the stability test of international pharmaceuticals guideline [24]. For the centrifugation tests, 3 g of each sample were centrifuged in triplicate for three cycles of 30 min at 3000 rpm. Sensorial features of the samples were examined at the same temperature, lighting and packaging conditions to assess variations in appearance, phase separation, color and odor.

### 4.4. Spreadability

The spreadability was evaluated by using a glass plate mold with a central circular orifice (diameter = 12 mm). The plate mold was placed on a supporting glass plate with a piece of graph paper and a light source underneath. The glass plate mold orifice was then completely filled with sample, and after leveling the sample surface, the plate mold was carefully removed and a glass plate of predetermined weight was put on the sample. After 1 min, the surface covered with sample was measured through the diameter in two orthogonal directions with the aid of the graph paper, and the medium diameter was calculated. This procedure was repeated adding new plates at 1-min intervals until the measured diameters did not show any perceptible variation [25] The spreadability was calculated according to Equation (1):

(1) $$Si=\frac{d^{2}\pi }{4}$$

where *Si* (mm^2^) is the spreadability for each weight and *d* is the mean diameter (mm).

The value of *Si* obtained in the limit where a subsequent addition of weight caused no significant change in the area of the supporting plate covered by sample was taken as the maximum spreadability. The stress limit was calculated based on the weight that led to the maximum spreadability. All samples were also evaluated using standard criteria for spreadability and acceptability [26]. Spreadability of samples was determined according to the criteria outlined in Table 3. Vehicles rating at “0” or “1” were considered to have adequate spreadability. All the analyses were made three times at room temperature (~25 °C).

### 4.5. pH Measurement

To determine the pH of the plant extract and hydrogel, a 10% *w*/*v* solution was prepared by adding 10 mg of each sample to 100 mL of distilled water. A digital pH meter (Model PHS-3B, PHTEK, São Paulo, Brazil) was used to read the pH of solutions directly. The pH meter was calibrated by testing the reading of buffer solutions with known pH at room temperature. pH 7 and 4 were used for the calibration and then the electrode on an adjustable arm was placed in the sample solutions.

### 4.6. Viscosity

Rheological analysis was performed on a Brookfield rotational viscometer (LV Model DV-E; spindle S62, Middleboro, MA, USA) with a coaxial cylinder sensor. The flow behaviour was studied by continuous shear investigations, which were performed to evaluate the shear stress (cP) as a function of shear rate (s^−1^). The study started with a shear rate of 0.05 s^−1^ up to a maximum of 5 s^−1^ and back to 0.05 s^−1^, and the resulting shear stress was measured. To reduce the influence of temperature on the rheological behaviour, the samples were maintained at 25 ± 1 °C during all experiments.

### 4.7. Gel Disintegration Study

Disintegration of hydrogels was examined by using tablet-disintegration apparatus (Model 298, Nova Ética). Hydrogels (0.5 g) were placed in the basket of disintegration apparatus having 900 mL of ultrapure water, maintained at 37 °C. The time taken for the complete disintegration of the hydrogels in ultrapure water was considered as the disintegration time.

### 4.8. Microbiological Analysis

Microbiological analysis was performed by the total viable count method (bacteria and fungi) and search for specific pathogens (*Pseudomonas aeruginosa*, *Staphylococcus aureus*). The methodology and acceptance criteria followed the specifications of the US Pharmacopoeia [27], recommended for non-sterile pharmaceutical products.

### 4.9. HPLC-DAD Analysis

Extractions of hydrogel samples (0.75 mg) were carried out with methanol (50 mL) using volumetric flasks with the aid of an ultrasonic bath for 40 min. The methanol extracts were filtered and concentrated using vacuum rotatory evaporation (85-01 LABTEC LB) at 40 °C. Methanol-free extracts were solubilized in distilled water, frozen at −10 °C and lyophilized (Liotop L108, Liobras, São Carlos, Brazil). Freeze-dried samples were prepared to a concentration of 1 mg/mL, with injection volume of 10 μL. Before injection, the samples were filtered through a 0.45 μm nylon membrane filter (Millipore, Billerica, MA, USA), and submitted to HPLC-DAD analysis. The apparatus used in the study consisted of a Shimadzu controller (CBM-20A); solvent pump unit (LC-20AT) and a Diode Array Detector (SPD-M20A) (all Shimadzu). UV spectra set at 254 nm were recorded for peak characterization. Separation was performed with a C18 column (Dr. Maisch GmbH; 5 μm, 250 mm × 4.6 mm; 25 °C). Mobile phase consisted of an isocratic system (A) 0.1% (*v*/*v*) aqueous solution of phosphoric acid and (B) acetonitrile (80:20); flow rate of 1 mL/min.

Punicalin and punicalagin analytical reference solutions were prepared by serial dilutions from methanol mother standard solutions of 1 mg/mL. The analytical curves were established using externally prepared standards, and five points from the standards solutions in methanol at concentrations of 0.5; 0.25; 0.125; 0.0625 and 0.0362 mg/mL, prepared on the same day on which the analyzes were performed. All analyzes were performed in triplicate.

### 4.10. Statistical Analysis

Results are presented as mean ± standard error of mean (SEM) and statistical significance between groups was determined by two-way analysis of variance (ANOVA) with Tukey-test post hoc analysis and Bonferroni (test of sphericity) for comparison of differences between data sets. *p*-Values less than 0.05 were considered to be significant.

## 5. Conclusions

The results of long-term and accelerated stability studies at 0, 3, 6 months showed that the ionic hydrogel formulation maintained a proper viscosity, suitable spreadability, no significant pH changes and microbiological quality within the recommended parameters. The ellagitannin punicalagin was found to be a stable biomarker, showing average yields ranging from 10.8 to 9.8 mg per 100 g of hydrogel under stressed storage conditions. Our results support that punicalagin standardized bioactive extracts of *P. granatum* can be formulated into a stable semi-solid dosage form. Formulations containing not less than 0.49% total punicalagins, determined by liquid chromatography and calculated with reference to a 2% (*w*/*w*) ethanolic extract hydrogel-based formulation might find good use in wound healing therapy. This formulation showed an excellent prognosis for its application as a therapeutic alternative in wound healing treatment, and is therefore suitable for future clinical studies. This is the first report of a case study using a standardized formulation of *Punica granatum* in wound healing treatment.

## Figures and Tables

**Table 1:** pH of hydrogel-based formulation in different storage conditions.

pH (Mean Values ± S.D ^1^)			
Storage Conditions	T0	T3	T6
-	4.95 ± 0.06	-	-
temperature test chamber—aluminum tube	-	4.84 ± 0.15	5.27 ± 0.08
climate chamber—aluminum tube	-	5.5 ± 0.12	5.49 ± 0.12
temperature test chamber—polyethylene tube	-	4.32 ± 0.10	5.27 ± 0.09
climate chamber—polyethylene tube	-	4.14 ± 0.08	4.18 ± 0.13

^1^ S.D: Standard deviation.

**Table 2:** Determination of significance levels when comparing correlations between PGHF, biomarkers, time and storage conditions after ANOVA.

	Correlations	PG	PN
T3	TTC × CC	0.941	0.046
T6	TTC × CCC	0.965	0.027
TTC	T3 × T6	0.057	0.057
CC	T3 × T6	0.126	0.069

PG, punicalagin; PN, punicalin; T3, time: 3 months; T6, time: 6 months; TTC, Temperature test chamber (30 °C, ambient humidity); CC, Climate chamber (40 °C, 75% RH).

**Table 3:** Criteria used to assess the spreadability of formulations

Score	Description
−2	Formulation appears as a solid state, which cannot be spread around the application site.
−1	Formulation has to be applied with force greater than normal to achieve a desirable spread.
0	Formulation displays good spreadability with desirable stay-on semisolid property.
1	Formulation displays good spreadability with a slight tendency to flow over the skin surface.
2	Formulation has low viscosity with high tendency to flow over the skin surface.
